# An Experimental Investigation into Trochoidal Milling for High-Quality GFRP Machining

**DOI:** 10.3390/ma18071669

**Published:** 2025-04-05

**Authors:** Ondřej Bílek, Martin Řezníček, Andrzej Matras, Tomáš Solařík, Lubomír Macků

**Affiliations:** 1 Department of Production Engineering, Faculty of Technology, Tomas Bata University in Zlín, Vavrečkova 5669, 76001 Zlín, Czech Republic; mreznicek@utb.cz (M.Ř.); t_solarik@utb.cz (T.S.); 2 Department of Production Engineering, Faculty of Mechanical Engineering, Cracow University of Technology, 31-155 Cracow, Poland; andrzej.matras@pk.edu.pl; 3 Department of Process Control, Faculty of Informatics, Tomas Bata University in Zlín, Nad Stráněmi 4551, 76005 Zlín, Czech Republic; macku@utb.cz

**Keywords:** GFRP, trochoidal milling, adaptive milling, composite machining, tool wear, cutting forces, surface roughness, burr formation, hybrid strategy, anisotropic materials

## Abstract

This study investigates the effectiveness of trochoidal (adaptive) milling in machining Glass Fiber Reinforced Polymer (GFRP), emphasizing its potential advantages over conventional milling. Six coated solid carbide end mills, each with distinct geometries, were evaluated under identical conditions to assess the cutting forces, surface quality, dimensional accuracy, burr formation, chip size distribution, and tool wear. Trochoidal milling demonstrated shorter cycle times—up to 23% faster—and higher material removal rates (MRRs), while conventional milling provided superior dimensional control and smoother surfaces in certain fiber-sensitive regions. A four-tooth cutter with a low helix angle (10°) and aluminum-oxide coating delivered the best overall performance, balancing minimal tool wear with high-quality finishes (arithmetic mean roughness, Ra, as low as 1.36 μm). The results indicate that although conventional milling can exhibit a 25%-lower RMS cutting force, its peak forces and extended machining times may limit the throughput. Conversely, trochoidal milling, when coupled with an appropriately robust tool, effectively manages the cutting forces, improves the surface quality, and reduces the machining time. Most chips produced were less than 11 μm in size, highlighting the need for suitable dust extraction. Notably, a hybrid approach—trochoidal roughing followed by conventional finishing—offers a promising method for achieving both efficient material removal and enhanced dimensional accuracy in GFRP components.

## 1. Introduction

Composite materials are engineered materials made from two or more constituent materials with significantly different physical and chemical properties. This combination results in a material with enhanced properties compared to the individual components [1,2]. Composite materials are primarily classified into the following two categories: matrix composites and particulate composites. Matrix composites consist of a continuous phase (matrix) and a dispersed phase (reinforcement), whereas particulate composites are characterized by particles embedded within a matrix [3,4]. The matrix can be polymeric, metallic, or ceramic, and the reinforcement can include fibers, particles, or flakes, each contributing to the overall mechanical properties of the composite [5,6]. The versatility of composites allows them to be tailored for various applications, ranging from aerospace to civil engineering.

Glass Fiber Reinforced Polymers (GFRPs) are notable among composite materials for their exceptional properties and advantages. One of the primary benefits of GFRP is its high strength-to-weight ratio, which allows for lightweight structures without compromising strength, making it ideal for applications in the aerospace and automotive industries [7,8]. Additionally, GFRP exhibits superior corrosion resistance compared to traditional materials such as steel, enhancing durability and reducing maintenance costs [9,10]. GFRP also offers high specific stiffness and fatigue resistance, crucial for long-term structural applications [8,11]. Furthermore, GFRP is nonmagnetic and has low thermal conductivity, making it suitable for environments where electromagnetic interference is a concern [10].

Despite these advantages, machining GFRP presents specific challenges owing to its unique material properties. The anisotropic nature of GFRP, where mechanical properties vary with fiber orientation, can lead to inconsistent machining outcomes, such as uneven surface finishes and varying tool wear rates [12,13]. The presence of both the polymer matrix and glass fibers complicates the cutting process because the tool encounters materials with different hardness and thermal properties, leading to issues such as fiber pull-out, delamination, and thermal radation during machining [13,14,15]. Additionally, the high abrasiveness of glass fibers contributes to rapid tool wear, necessitating frequent tool replacement and increasing production costs [12].

The machinability of GFRP differs significantly from that of traditional metallic materials. Metals typically exhibit isotropic properties, resulting in more predictable machining outcomes, whereas the anisotropic and heterogeneous properties of GFRP can lead to inconsistent cutting forces and surface finishes [16,17]. Moreover, metals generally have uniform material properties and do not suffer from fiber-related complications, such as delamination and fiber pull-out [11,18]. The thermal properties of GFRP also complicate machining, as excessive heat generated during cutting can degrade the polymer matrix, affecting the integrity of the material [19]. Consequently, machining GFRP requires careful consideration of cutting parameters, such as speed and feed rate, to minimize damage and achieve the desired surface quality [20].

Conventional machining methods used for GFRP include milling, drilling, and grinding. However, these techniques have limitations owing to the anisotropic and abrasive characteristics of the material. Milling can lead to inconsistent cutting forces and increased tool wear [12,20]. Drilling poses challenges such as high surface roughness, delamination, and thermal damage if the parameters are not carefully controlled [19,21]. Grinding often results in high tool wear and excessive heat generation, which may degrade the polymer matrix [22].

To overcome these challenges, advanced machining strategies such as High-Efficiency Milling (HEM), trochoidal milling, and adaptive milling have been developed. HEM focuses on maximizing material removal rates while minimizing tool wear and heat generation using high spindle speeds, high feed rates, low radial engagement (stepover), and high axial engagement (stepdown) [23,24]. Trochoidal milling involves moving the cutting tool in a series of small overlapping circular (trochoidal) paths as it progresses along the desired toolpath. This technique allows for consistent and reduced radial engagement of the tool with the material, thereby reducing the cutting forces and heat generation [25,26]. Adaptive milling, on the other hand, is a CAM (Computer-Aided Manufacturing) software strategy that dynamically adjusts the toolpath to maintain a constant cutting load, adapting to the geometry of the workpiece to ensure optimal tool engagement throughout the machining process.

While all three techniques aim to enhance machining efficiency and tool longevity, they do so through different strategies. The HEM adjusts cutting parameters for overall efficiency, focusing on balancing speeds, feeds, and depths of cut. Trochoidal milling modifies the tool’s movement pattern to reduce stress by employing specific motion patterns that minimize tool engagement and cutting forces, making it ideal for challenging cuts such as deep slots or hard materials [27,28]. Adaptive milling leverages advanced CAM software algorithms to create intelligent toolpaths that adapt to the workpiece shape, maintain a constant cutting load, and optimize the machining process for complex geometries [29]. Despite their differences, these methods are often interrelated and can be used in conjunction to address the specific challenges of machining composites such as GFRP.

These advanced machining strategies offer several benefits for machining composites. Trochoidal milling, for instance, can reduce cutting forces by up to 70% compared to conventional methods [26,30], improve tool life, enhance surface quality, and allow for higher cutting speeds and feeds owing to better heat management. The intermittent cutting action in trochoidal milling allows the tool to cool between engagements, prolonging tool life, and enhancing chip evacuation [27,31]. Adaptive milling strategies can lead to improved accuracy and surface roughness by dynamically adjusting tool paths and cutting conditions based on the material response during machining [32]. This is particularly beneficial for machining complex geometries and dealing with variable material volumes.

Recent research on HEM and trochoidal milling of GFRP has highlighted the importance of optimizing cutting parameters to enhance machining outcomes. Studies indicate that feed rate significantly influences surface roughness, and careful selection of cutting parameters is essential to achieve the desired surface quality while minimizing defects [33]. Research utilizing the Taguchi method has identified the optimal cutting parameters to reduce delamination during the drilling and milling processes [34]. Moreover, adaptive feed-rate scheduling strategies have been proposed to enhance the efficiency of trochoidal milling, allowing for adjustments based on real-time conditions [35].

Different cutting parameters influence the machining outcomes of GFRP. The cutting speed can reduce cutting forces and improve surface quality up to a certain threshold, beyond which excessive speeds may cause thermal degradation [36,37]. The feed rate is critical, as higher feed rates increase the likelihood of delamination owing to greater forces [38]. The depth of cut impacts the cutting forces and thermal effects, necessitating a balance to prevent fiber pull-out and delamination [39]. Tool geometry and material play crucial roles, with optimized geometries reducing cutting forces and improving the surface finish [40,41].

Studies comparing the effectiveness of various tools in trochoidal milling or HEM of GFRP have demonstrated that the tool material and geometry significantly influence the machining quality and efficiency. Poly-Crystalline Diamond (PCD) tools exhibit superior performance in terms of feed force and surface finish compared with uncoated carbide tools [42]. Specialized trochoidal milling tools can provide better surface quality and reduced delamination [43].

Nevertheless, trochoidal milling has several drawbacks that must be considered. The continuously curved toolpath can introduce additional travel distance, potentially prolonging the cycle time if the parameters are not carefully optimized [44]. Furthermore, advanced variants such as adaptive trochoidal milling often require high-performance machine tools and sophisticated CNC controls, along with advanced CAM software necessary to generate these complex trajectories, thereby elevating implementation costs and limiting use in less-equipped manufacturing environments [30]. Finally, the intermittent cutting action—although effective at mitigating heat and force—may not fully eliminate challenges such as fiber pull-out or delamination in heterogeneous materials unless process conditions and tool design are precisely tuned to the composite’s anisotropic nature [45].

A parallel can be drawn between the study of hygrothermal aging in carbon-fiber/epoxy composites modified by nylon-6 and the present work on optimizing milling strategies for GFRP. The study by Xian [46] underscores how water ingress, elevated temperatures, and interface degradation can critically diminish mechanical performance over time. Such insights into the vulnerability of fiber-reinforced polymers under environmental stresses reinforce the importance of careful process control during machining. In particular, choosing an effective milling strategy—such as the trochoidal approach evaluated here—may minimize local thermal spikes and mechanical stresses that could otherwise exacerbate microcracking and weaken fiber/matrix bonds. Conversely, ensuring robust interfacial stability, as highlighted by hygrothermal research, stands to maintain the dimensional accuracy and surface quality necessary for high-value composite components processed under demanding industrial conditions.

Factors influencing the cutting forces during the machining of GFRP include cutting speed, feed rate, depth of cut, tool geometry, fiber orientation, temperature, and tool wear. Understanding and optimizing these parameters is crucial for improving the machining efficiency and surface quality [39,47].

The cutting conditions and machining strategies significantly affect the surface quality of the GFRP material. Higher cutting speeds can improve surface quality by reducing cutting forces; however, excessive speeds may generate heat that damages the polymer matrix [37,48]. The feed rate influences the surface roughness, with higher feed rates leading to poorer surface quality owing to higher cutting forces [49,50].

The types of chips produced when machining GFRP, such as continuous, serrated, brittle, or dust-like chips, provide insights into the cutting process. Continuous chips indicate optimal cutting conditions, whereas serrated or brittle chips suggest the need for parameter adjustments [48,51]. Health risks associated with machining GFRP, especially concerning dust particles and fibrous fragments, include respiratory issues, potential cancer risks, dermal exposure, chronic health effects, and increased susceptibility to acute respiratory infections [52,53]. As summarized in Table 1, machining composites such as CFRP (Carbon Fiber Reinforced Polymer) and GFRP generates predominantly respirable dust particles, often smaller than 10 μm, with some fiber fragments measuring as small as 0.05–0.1 μm in diameter [54,55,56,57,58,59]. These fine airborne particles can penetrate deep into the lungs, posing the risk of fibrosis, chronic inflammatory responses, and potential carcinogenic effects, similar to asbestos exposure [55,58].

To mitigate these risks, effective dust extraction systems must be employed, including high-efficiency particulate air (HEPA) filtration, local exhaust ventilation (LEV), and industrial vacuum systems with fine particle capture capabilities [55,56]. The encapsulation of machining zones and wet machining methods can further minimize airborne dust dispersion. In addition to engineering controls, personal protective equipment (PPE) such as respirators (FFP2/FFP3 or N95/P100), protective eyewear, and disposable overalls, are essential for worker safety [55,58,59].

Regulatory frameworks addressing composite dust exposure include OSHA (Occupational Safety and Health Administration) standards, NIOSH (National Institute for Occupational Safety and Health) guidelines, and EU directives on workplace exposure limits for airborne particulates. Although no specific CFRP/GFRP dust exposure limits exist globally, general dust exposure thresholds (e.g., OSHA PEL: 5 mg/m^3^ respirable dust, 15 mg/m^3^ total dust) have been applied, and stricter recommendations (e.g., NIOSH REL: 3 fibers/cm^3^) have been proposed for airborne fibers owing to their potential health hazards [54,55].

Trochoidal milling and HEM affect productivity and manufacturing efficiency by increasing material removal rates, reducing cutting forces, improving surface quality, and enabling adaptive feed rate scheduling [23,60]. These strategies enhance the machining of GFRP and contribute to cost savings and manufacturing capabilities.

The newest technological innovations in the field of machining composite materials include advanced tooling technologies such as PCD tools, hybrid machining techniques such as ultrasonic vibration-assisted machining, adaptive machining strategies, smart manufacturing technologies with monitoring and sensing systems, and sustainable machining practices such as dry and minimal quantity lubrication techniques [3,61]. New machining methods being developed, such as adaptive milling strategies in CAM systems, aim to improve quality and efficiency when working with composites. Adaptive strategies in CAM systems can improve the accuracy and surface roughness by dynamically adjusting the tool paths and cutting conditions [29]. The integration of machine learning techniques helps predict material behavior and optimize cutting parameters, leading to enhanced machining outcomes [32].

In this study, we address the specific challenges of GFRP machining by examining how advanced trochoidal milling strategies interact with a diverse set of tool geometries under both trochoidal and conventional strategies. By comparing six end mills, we provide a data-driven perspective on critical output parameters—cutting forces, dimensional accuracy, surface finish, burr formation, tool wear, and dust generation—while also incorporating an economic cost function that highlights overall productivity. This approach enables us to elucidate the complex interplay between anisotropic fiber reinforcement, process settings, and tool characteristics, thereby filling important knowledge gaps in composite machining.

## 2. Materials and Methods

This study conducted a comprehensive investigation of six cutting tools specifically selected for their suitability in machining Glass Fiber Reinforced Polymer (GFRP) composites. The experimental work encompassed both conventional and trochoidal milling methods, utilizing these tools to perform machining operations on a Computer Numerical Control (CNC) machining center. To facilitate machining operations, machining programs were generated using standard CNC programming methodologies. Siemens NX software (version 12) was employed for the programming tasks, providing a robust and versatile environment to accurately define the CNC machine movements and tool actions. Specifically, the Adaptive Milling function within NX was utilized, which allows dynamic adjustment of tool paths to maintain consistent cutting conditions.

By integrating the selected cutting tools with CNC machining technology and advanced programming via NX software, a systematic and controlled approach was established to investigate the machining characteristics and performance of the GFRP composite. The primary objectives of the experiment were to evaluate the effectiveness of these tools in addressing the challenges posed by the anisotropic and heterogeneous nature of GFRP, and to optimize the machining parameters for enhanced material removal rates and surface finish quality.

### 2.1. Cutting Tools

In this study, six solid carbide end mills from Seco Tools were selected based on their technical specifications suitable for machining GFRP composites. The specifications of these cutting tools are listed in Table 2. Each end mill has a cutting diameter of 6 mm and features coated cutting edges to enhance performance and tool life. Five of the tools were coated with DURA (CVD aluminum-oxide) coating, which is known for its exceptional hardness and wear resistance, while tool T6 was coated with NXT (TiAlN), providing high thermal stability.

During tool clamping in the collet chuck, careful attention was paid to maintaining a constant overhang length to ensure consistent machining conditions. Prior to each machining operation, radial and axial tool offsets were measured and compensated for, contributing to precise and accurate machining outcomes.

**Figure 1:**
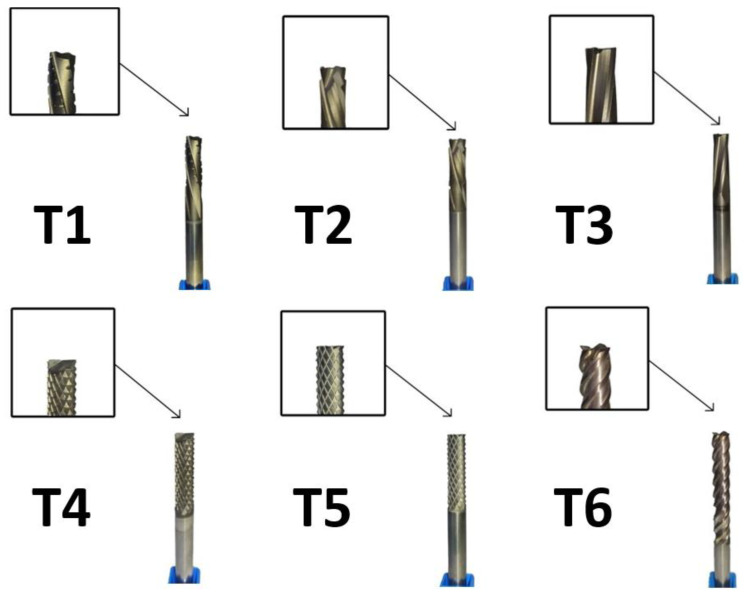
Photographs of the six cutting tools used in this study (from left to right: T1 to T6).

The selected cutting tools exhibited unique design features tailored for machining composite materials, particularly GFRP. The specific tools used in this study are depicted in Figure 1 and are described as follows:

- Tool T1—Honeycomb-Structured Cutter: Designed specifically for slot and side milling of sandwich-like composite materials, T1 features a unique honeycomb structure with sharp cutting edges and regular chip breakers on all five sides. Its left-handed spiral with a 15° helix angle induces a downward axial force (*Fz*) during cutting, enhancing chip evacuation, and reducing delamination;
- Tool T2—Compressive Cutter with Dual Opposing Helix Angles: T2 is engineered for slot and side milling, incorporating a compressive design with two counter-rotating helix angles set at ±20°. This configuration stabilizes the machining process and minimizes delamination, making it highly effective for layered composite materials;
- Tool T3—Four-Tooth Cutter for Thermosetting and Thermoplastic Composites: Specifically designed for slot and side milling, T3 integrates four teeth with a corner radius of 0.2 mm. It has the lowest flute helix angle among the tools, which is advantageous for achieving better surface quality during finishing operations;
- Tool T4—Multi-Tooth Composite Router with Two Face Cutting Edges: This versatile T4 cutter is suitable for side, slot, ramping, and straight machining operations. Equipped with two cutting edges that extend through the center, it demonstrates superior machining capabilities on composite material surfaces, particularly on the bottom plane;

- Tool T5—Multi-Tooth Composite Router with Ten Face Cutting Edges: Similar to T4, T5 is designed for side, slot, ramping, and straight machining, but features ten face cutting edges. This increases the material removal rates and allows for efficient high-volume machining;
- Tool T6—Universal Corner Milling Cutter with Stable Coating: Serving as a reference tool, T6 is intended primarily for side milling with an impressive axial cutting depth of up to 35 mm. It has a stable NXT (TiAlN) coating, ensuring consistent cutting performance and contributing to the overall stability of the machining process.

The selection of these cutting tools is instrumental in optimizing the machining process for GFRP composites. The DURA and NXT coatings enhance tool wear resistance and prolong tool life, ensuring efficient material removal with minimal tool wear. By monitoring the tool setup and incorporating appropriate compensations, this study aims to achieve high-quality machining outcomes, ultimately contributing to advancements in composite material processing techniques.

Economically, T1 was the most expensive tool among the selections, with a declining cost trend observed for ascending tool numbers. Interestingly, T4 and T5, despite differing in the number of cutting edges, had equivalent costs. The cost difference between the most expensive tool (T1) and least expensive tool (T6) was approximately 27%. This cost analysis is crucial for practical applications where tool performance must be balanced against economic considerations.

### 2.2. GFRP Material

For the machining experiments, a GFRP material was selected and meticulously prepared to simulate practical machining applications, such as aperture and pocket fabrication. The GFRP material, originally in the form of a spring component, was chosen owing to its relevance in industries where such geometries are common, notably in the aerospace and automotive sectors.

The GFRP spring was composed from a unidirectional prepreg combined with a DT806 epoxy matrix, a group of low-to-medium-viscosity epoxy resins known for their excellent mechanical properties and suitability for composite manufacturing. The cured neat resin exhibited a density of 1.21 g/cm^3^, indicating a robust matrix. The epoxy matrix was sourced from Delta-Preg (Sant’Egidio alla Vibrata TE, Italy), which is a supplier of advanced composite materials.

The unidirectional prepreg used in the GFRP material featured a plain-weave fabric with a surface weight of 425 g/m^2^. The fiber content had a weight ratio of 90% in the *X*-direction and 10% in the *Y*-direction, providing anisotropic mechanical properties characteristic of GFRP composites. The fibers used were EC95 × 136 tex and EC9 68 tex types, known for their high strength and stiffness, which contribute significantly to the overall mechanical performance of the composite.

**Figure 2:**
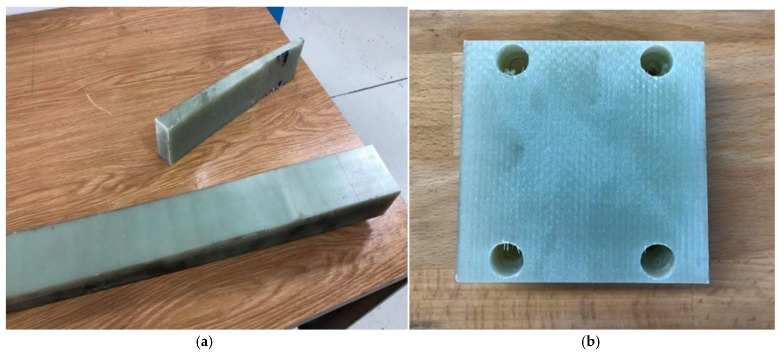
(**a**) GFRP spring in partially divided state; (**b**) prepared GFRP block with clamping holes.

To prepare the material for machining, the GFRP spring was initially sectioned into blocks with approximate dimensions of 76 × 76 × 40 mm using a precision disc saw, as depicted in Figure 2a. This size was selected to accommodate the machining operations and ensure secure mounting on the dynamometer. Four through-holes were drilled along the periphery of each workpiece, complete with countersinks, to facilitate attachment to the dynamometer mounting plate. This setup was essential for the accurate measurement of cutting forces during the experiments.

Within the central region of each GFRP block, an oblong pocket measuring 60 mm × 46 mm × 12 mm was machined during the experiments (Figure 2b). The pocket featured a corner radius of 4 mm, replicating the typical features found in industrial components. This design allowed for the assessment of the cutting tools’ performance in producing precise internal geometries and surface finishes on GFRP materials.

The selection and preparation of this specific GFRP material aimed to replicate real-world machining scenarios and address the challenges associated with machining anisotropic and heterogeneous composite materials. The unidirectional fiber orientation and properties of the DT806 epoxy matrix provided a suitable test material for investigating the effects of different cutting tools and machining strategies, particularly trochoidal milling, on surface quality, tool wear, and overall machining efficiency.

### 2.3. Experimental Setup

**Figure 3:**
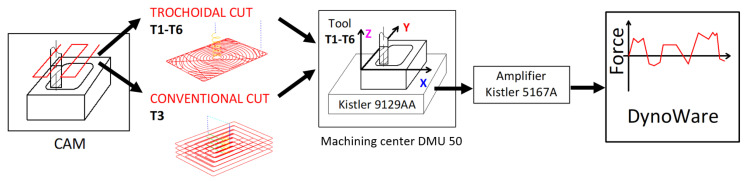
Schematic of the experimental setup with measurement of cutting forces.

The experimental investigation was designed to evaluate the performance of six selected cutting tools (T1–T6) in machining GFRP materials using both trochoidal (adaptive) and conventional milling strategies. The workpiece and tools detailed in the preceding sections formed the foundational elements of this study. The experimental setup is schematically depicted in Figure 3.

**Figure 4:**
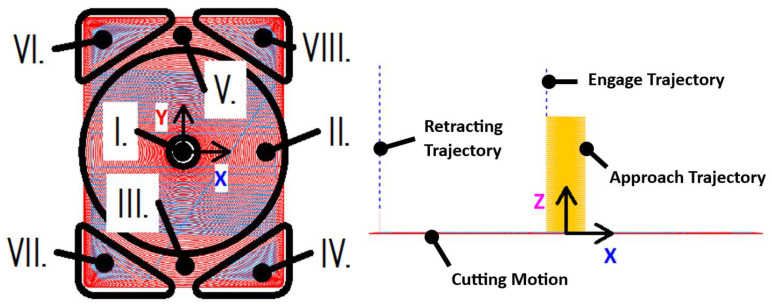
Tool paths with stages during trochoidal milling: plan view (**left**); side view (**right**).

**Figure 5:**
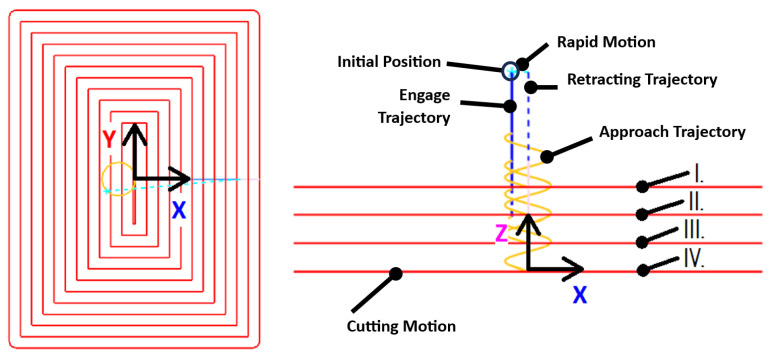
Tool paths during conventional milling: plan view (**left**); side view with stages of cutting levels (**right**).

The initial step involved the development of a machining program to fabricate a rectangular pocket with dimensions suitable for assessing the machining performance (Figure 4 and Figure 5). Using Siemens NX CAM software (version 12), two distinct machining strategies were programmed:

- Trochoidal (adaptive milling): This strategy employs the Adaptive Milling function in NX to generate a trochoidal tool path that maintains a consistent cutting load by dynamically adjusting the tool engagement. The trochoidal milling process involves cutting the material radially within an extremely narrow width, constituting 7% of the tool diameter (0.42 mm), with an axial depth of cut of 12 mm (200% of the tool diameter);
- Conventional: The conventional strategy utilized the Cavity Mill function in NX, dividing the machining process into four distinct cutting levels. The radial depth of cut was set to 30% of the tool diameter (1.8 mm), and the axial depth of cut varied from 1.5 mm (Stage I) to 3.5 mm (Stages II–IV).

The CAM software calculated the machining times as 3 min and 49 s for trochoidal milling and 5 min and 26 s for conventional milling. The machining parameters for both strategies are summarized in Table 3.

The tool paths for trochoidal milling are shown in Figure 4. The machining program was uniform across all tools (T1–T6), starting with an initial helical entry (Stage I) down to the pocket bottom. This was followed by peripheral milling towards the nearest walls in spiral motion (Stage II) and incremental trochoidal milling of residual material within islands (Stages III and V) as well as corners (Stages IV, VI, VII, and VIII). The cutting speed was set to 220 m/min with a feed rate of 2652 mm/min, resulting in a high material removal rate (MRR) of 13.37 cm^3^/min.

Figure 5 shows the tool paths for conventional milling. The radial depth of the cut was increased to 30% of the tool diameter, and the cuts were less frequent compared with trochoidal milling. The pocket was machined across multiple levels, with the first level (Stage I) at a depth of 1.5 mm and subsequent levels (Stages II–IV) at a 3.5 mm depth of cut. The cutting speed was reduced to 145 m/min, and the feed rate was set to 1000 mm/min.

**Figure 6:**
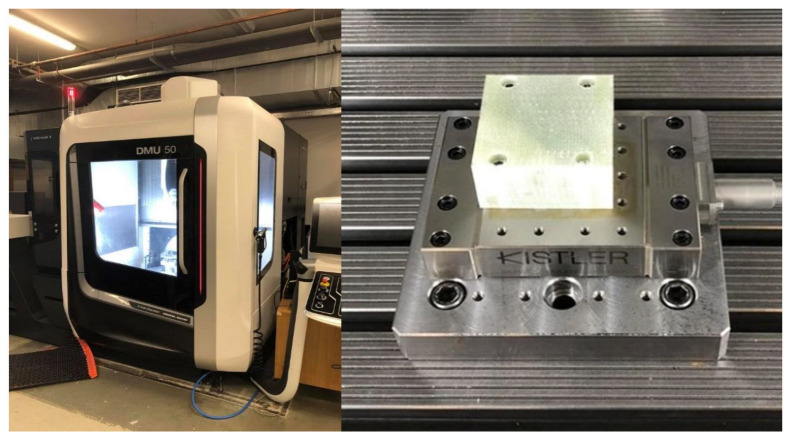
DMG Mori DMU 50 3rd Generation CNC machining center (**left**); GFRP workpiece clamped on a Kistler 9129AA dynamometer (**right**).

Machining experiments were conducted on a DMG Mori DMU 50 3rd Generation CNC machining center, Bielefeld, Germany (Figure 6, left). The GFRP workpiece was securely mounted on the machine table using a piezoelectric dynamometer (Kistler 9129AA), Winterthur, Switzerland designed to measure the cutting force components along the *X*-, *Y*-, and *Z*-axes. The workpiece was clamped onto the mounting surface of the dynamometer using four screws, ensuring stability during machining (Figure 6, right).

The dynamometer signal was transmitted to a Kistler 5167A amplifier and then to a computer equipped with the Kistler Dynoware software (version 3.1.0.0). This setup enabled real-time recording and analysis of the cutting force components (*Fx*, *Fy*, and *Fz*) aligned with the Cartesian coordinate system of the machine.

Cutting forces are critical indicators of tool performance, machinability, and deformation behavior during machining processes. They directly influence the tool deflection, surface finish, dimensional accuracy, and tool wear. In milling operations, the lateral cutting forces (*Fx* and *Fy*) contribute to tool deflection owing to bending, which can adversely affect the surface quality and dimensional accuracy. The axial force (*Fz*) affects the penetration of the tool into the material and can influence the stability of the machining process. Understanding and analyzing these forces is essential for optimizing machining parameters and tool selection, especially when working with anisotropic and abrasive materials like GFRP composites [12,20].

**Figure 7:**
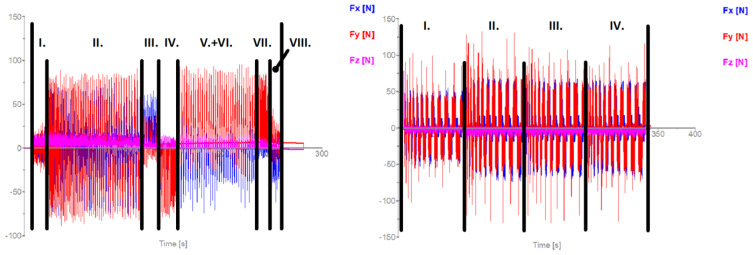
Recorded cutting force components for trochoidal milling (**left**); conventional milling (**right**) using tool T1.

The recorded cutting forces provide insights into the machining dynamics of each milling strategy. For trochoidal milling (Figure 7, left), the axial force component (*Fz*) was the least significant, with lateral forces (*Fx* and *Fy*) dominating based on the tool’s position. The effective machining time was 4 min and 11 s, representing a 9% increase from the CAM-calculated time owing to the execution of 2078 lines of G-code and machine tool dynamics.

In conventional milling (Figure 7, right), the cutting force record was subdivided into four stages corresponding to the varying depths of cut. The initial stage involved a shallower depth (1.5 mm) with more pronounced fluctuations in the lateral forces (*Fx* and *Fy*), which is characteristic of conventional machining without load-optimized strategies. The actual machining time matched the CAM estimate at 5 min and 27 s, involving 365 lines of G-code.

### 2.4. Evaluation Methodology

To assess the performance of the cutting tools and machining strategies, a comprehensive evaluation methodology was implemented, focusing on dimensional accuracy, surface quality, chip analysis, and tool wear.

**Figure 8:**
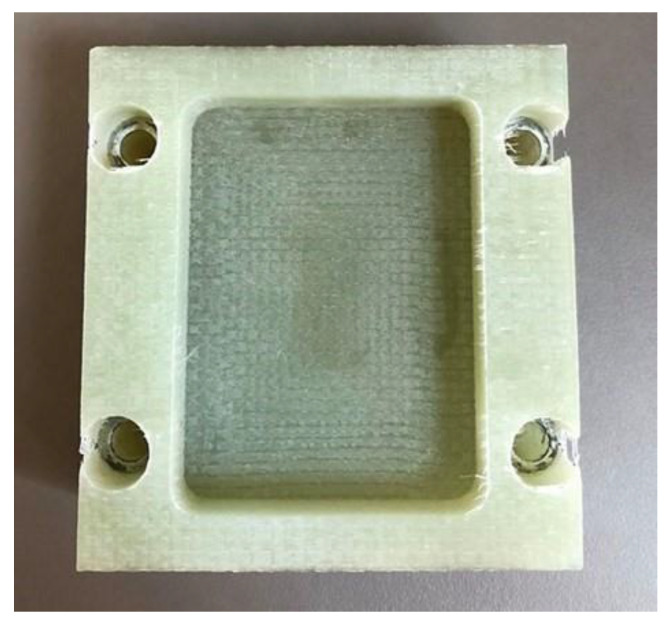
Workpiece after machining experiments.

**Figure 9:**
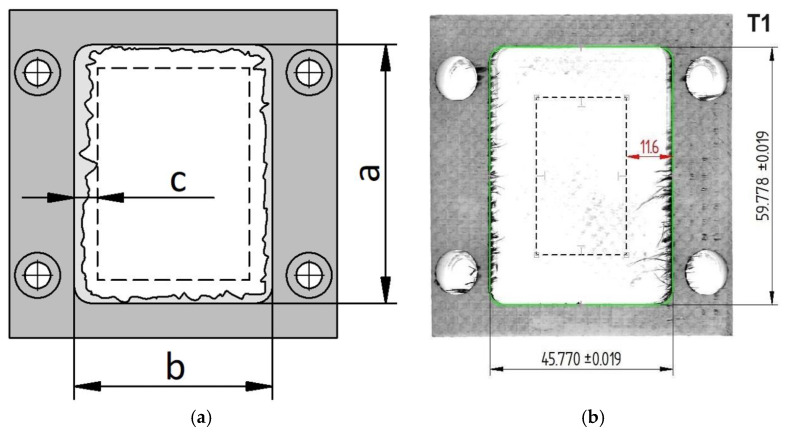
Dimensional deviations and burr measurement parameters: (**a**) schematic of measurement points; (**b**) illustration of burr measurement for tool T1.

After machining (Figure 8), the workpieces with machined pockets were subjected to dimensional inspection to measure deviations from the nominal dimensions and quantify burr formation (Figure 9). Measurements were conducted using a Mitutoyo digital caliper with a resolution of 0.01 mm. The parameters measured included the following:

- Transverse length of the pocket wall (a): measured along the *X*-axis;
- Longitudinal length of the pocket wall (b): measured along the *Y*-axis;
- Maximum burr size (c): determined by optical examination and digitization using a high-resolution scanner.

A statistical analysis was performed on the collected data to evaluate the consistency and accuracy of the machining process. The burr size is particularly important as it affects the quality and functionality of the machined component.

**Figure 10:**
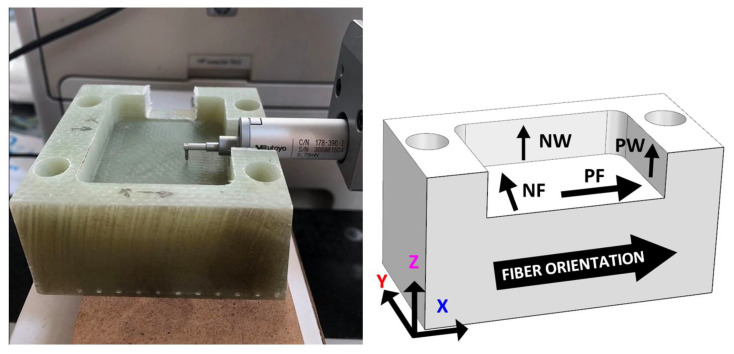
Measurement of machined surface quality (**left**); surface orientation relative to fiber direction (**right**). Symbol nomenclature: N—normal; P—parallel; W—wall; F—floor.

The quality of the machined surfaces was evaluated using a Mitutoyo SJ-410 tactile roughness tester, Kawasaki, Japan, adhering to ISO 4288 standards [62] for the measurement conditions (Figure 10, left). The profilometric parameters assessed were as follows:

- Arithmetic mean roughness (Ra): indicates average surface roughness.

Measurements were conducted at specified locations on the workpiece to account for the anisotropic nature of the GFRP material (Figure 10, right):

- Pocket floor—parallel to fiber direction (PF): measurements along the fiber orientation;
- Pocket floor—normal to fiber direction (NF): measurements perpendicular to the fiber orientation;
- Pocket wall—parallel wall (PW): short inner wall.
- Pocket wall—normal wall (NW): long inner wall.

Owing to the pocket depth, grooves were machined into the walls to facilitate probe access. Each measurement was repeated 14 times, resulting in 56 measurements per sample to ensure statistical significance. A comprehensive statistical analysis was performed, including the calculation of the mean (*μ)* and standard deviation (s), as well as the application of Grubbs’ test and other relevant statistical methods to identify outliers. The processed data were then graphically presented to highlight the trends and differences across the various cutting strategies. (See Appendix A Table A1 for a summary of Ra [µm] for different tools, measured areas, and cutting strategies.)

**Figure 11:**
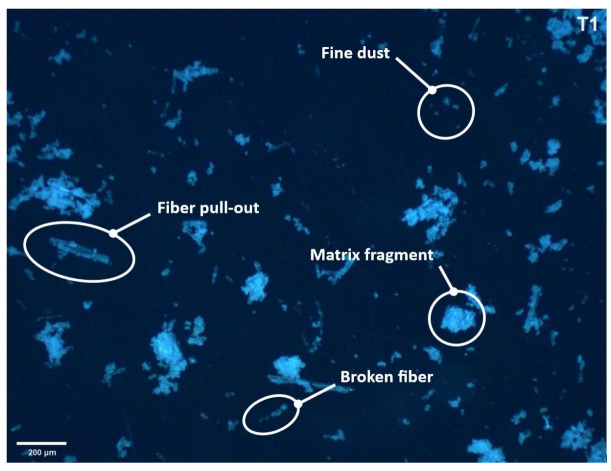
Photograph of chips after trochoidal milling using tool T1.

The chips produced during the machining process were collected and analyzed to determine the size distribution and morphology, which are indicative of the cutting mechanisms and tool performance. The chips were examined using a Leica DMI 3000M microscope (Leica Microsystems, Wetzlar, Germany) at 50× magnification (Figure 11). For a more detailed analysis, ImageJ (version 1.52u) was employed, providing semi-automated quantification of chip dimensions. After binarizing the microscope images, the software identified each chip as a distinct particle, enabling the calculation of particle area in pixels. Then, the maximum Feret diameter was utilized (i.e., the longest distance between any two points on the particle boundary) as our primary dimensional parameter, since it effectively represents the size of irregularly shaped and elongated chips and is directly relevant to chip transport and tool wear mechanisms. The resulting size distribution data were summarized in histograms using Microsoft Excel.

The chip analysis provided insights into the effectiveness of each cutting tool and the machining strategy for producing desirable chip forms, which is crucial for understanding the machinability of GFRP composites and for assessing the potential health risks associated with dust and fibrous particles.

Tool wear was evaluated as a critical factor in determining the suitability of cutting tools for machining GFRP. Before and after the machining experiments, each tool was examined using a HAIMER UNO 20/40 tool presetter (HAIMER, Wels, Austria) and measuring device. Wear patterns were documented, focusing on flank wear, crater wear, and edge chipping. The extent of tool wear provided insights into the durability and performance of each tool under specified cutting conditions and machining strategies.

All data collected from dimensional measurements, surface roughness assessments, chip analysis, and tool wear evaluations were statistically analyzed to identify significant differences between the tools and machining strategies. The root mean square (RMS) values of the cutting forces were calculated to quantify the overall machining load. In addition to the RMS cutting forces, the specific cutting force was also determined, providing a normalized measure of the cutting efficiency with respect to the cross-sectional area of material removal and enabling more accurate comparisons of the tool performance under varying cutting conditions.

By carefully designing the experimental setup and systematically applying evaluation methodologies, this study aims to advance the understanding of effective machining practices for GFRP composites. All the data were synthesized into a comprehensive overview table to determine the overall performance of each tool and guide the selection of optimal machining strategies.

## 3. Results

In this section, a summary and experimental findings are presented, focusing on six key criteria: cutting forces, surface quality of machined surfaces, dimensional accuracy of pocket geometry, burr formation, tool wear, and chip morphology analysis. These criteria were evaluated for both trochoidal and conventional milling strategies employed in the machining of GFRP composites.

### 3.1. Cutting Forces

The cutting forces were measured during the machining of the GFRP material using both trochoidal and conventional milling strategies with six different cutting tools (T1–T6). The cutting force data, including the root mean square (RMS) and maximum values, are summarized in Table 4.

In trochoidal milling, the cutting forces exhibited notable variations among the different tools. The maximum lateral forces (*Fx* and *Fy*) are critical, as they are directly related to tool deflection and potential deviations in the machined geometry. Tool T3 showed the highest maximum lateral forces, with *Fx* reaching 116.20 N and *Fy* peaking at 141.00 N. This can be attributed to T3’s tool geometry, specifically its lower flute helix angle and four-tooth design, which may lead to increased engagement with the workpiece material and higher cutting forces.

Conversely, tool T5, a ten-tooth router, demonstrated the lowest maximum lateral forces with *Fx* at 93.95 N and *Fy* at 90.69 N. The higher number of cutting edges in T5 likely distributed the cutting load more evenly, reducing the force on any single edge and resulting in lower overall lateral forces. This distribution can enhance machining stability and reduce tool deflection, positively impacting the surface quality.

The axial force (*Fz*) is also a significant parameter, particularly in the context of tool wear and potential delamination in composite materials. Tool T6 exhibited the highest maximum axial force at 101.80 N. T6, being a universal corner milling cutter with a stable NXT (TiAlN) coating and a high helix angle of 46°, may induce higher axial forces owing to the increased thrust during cutting. In contrast, tool T1 showed the lowest maximum axial force at 25.19 N, which can be attributed to its left-handed helix design with a 15° angle that induces a downward axial force, effectively reducing the axial load during cutting.

Examining the RMS values provides insight into the average cutting forces experienced during milling. The RMS values of lateral forces in trochoidal milling ranged from 19.09 N to 30.25 N, indicating a relatively narrow dispersion. Tool T3 had the lowest RMS value for *Fx* (19.09 N), whereas T6 had the highest RMS value for *Fy* (30.25 N). For the axial force *Fz*, T3 again exhibited the lowest RMS value at 4.70 N, suggesting that despite its high maximum forces, the average axial load was relatively low. T6 had the highest RMS value for *Fz* at 18.39 N, consistent with its high maximum axial force.

For conventional milling, the cutting forces were measured using tool T3 for comparison. The RMS values for *Fx* and *Fy* were 19.59 N and 24.96 N, respectively, which are comparable to the values observed in trochoidal milling. However, the maximum lateral force *Fy* reached 144.50 N, exceeding the maximum observed in trochoidal milling with T3. This suggests that conventional milling may lead to higher peak forces, potentially owing to the intermittent nature of tool engagement and larger radial depths of cut, which can cause sudden increases in the cutting load [33].

The RMS value for the axial force *Fz* in conventional milling with T3 was 4.60 N, slightly lower than that in trochoidal milling. This indicates that the average axial load was reduced in conventional milling, possibly owing to the smaller axial depths of cut used in this strategy.

The specific cutting force *kc* was calculated as the ratio of the resultant cutting force to the chip cross-sectional area. It provides a measure of the resistance of the material to cutting and is useful for comparing different machining conditions and tools. The *kc* values are presented in Table 4 for both the RMS and maximum forces.

During trochoidal milling, the effective specific cutting force *kc* remained relatively stable across the different tools, with RMS values fluctuating within a 15.2% range and maximum values within a 33.4% range. Tool T3 exhibited a significant drop in the specific cutting force when transitioning from trochoidal to conventional milling. The RMS *kc* value for T3 in conventional milling was 3.96 N/mm^2^, which is 25% lower than in trochoidal milling. This reduction suggests that conventional milling with T3 requires less force per unit area of the chip, indicating potentially better machinability under these conditions.

The analysis of cutting forces indicates that the choice of tool geometry and milling strategy significantly affects the machining performance of GFRP composites. Trochoidal milling is designed to maintain a consistent cutting load using a small radial depth of cut and higher feed rates, which can reduce the occurrence of sudden force spikes and improve tool life. However, the results show that certain tools, such as T3, may still experience high maximum forces in trochoidal milling, possibly owing to their specific geometrical features.

Tool T1’s reduced axial forces highlight the effectiveness of specialized tool designs in minimizing the cutting loads. The left-handed helix with a 15° angle in T1 induces a downward axial force that assists in chip evacuation and reduces the axial load on the tool, which is beneficial in machining layered composites such as GFRP. This aligns with findings in the literature [40,41,63] that suggest that helix angle and tool geometry play crucial roles in cutting force generation and tool performance.

The lower effective cutting forces observed in conventional milling with T3 suggest that under certain conditions, conventional milling may offer advantages in terms of reduced average cutting loads. However, the higher maximum forces and force fluctuations associated with conventional milling can lead to increased tool wear and potential damage to the workpiece owing to impact disturbances at the beginning of each cut. These force spikes can be detrimental when machining brittle and anisotropic materials such as GFRP, as they may exacerbate delamination and fiber pull-out.

The specific cutting force analysis reinforces the observation that conventional milling with T3 results in lower force requirements per unit area of the chip, indicating improved cutting efficiency. However, the tradeoff between lower average forces and higher peak forces must be carefully considered when selecting a machining strategy for GFRP materials.

The cutting force measurements indicate that both milling strategies have advantages and limitations when machining GFRP composites. Trochoidal milling, combined with appropriate tool selection, can lead to more consistent cutting forces and potentially better surface quality owing to reduced tool deflection. However, certain tools may still experience high maximum forces during trochoidal milling. The choice between trochoidal and conventional milling should be made based on a comprehensive assessment of the tool geometry, material properties, and desired machining outcomes.

### 3.2. Surface Quality

**Figure 12:**
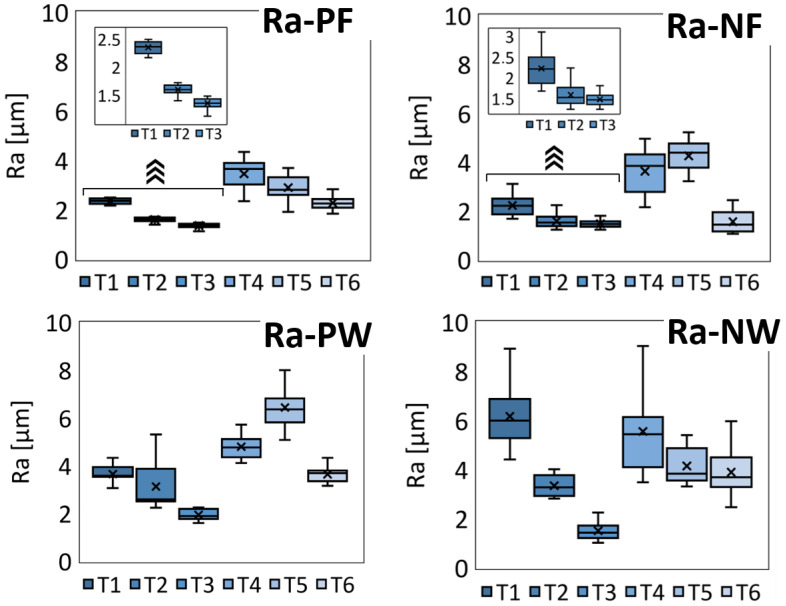
Box-and-whisker plots showing Ra values for the pocket floor (PF, NF) and internal walls (PW, NW) for all tools.

The surface quality in machining GFRP was evaluated based on arithmetic mean roughness (Ra) measurements, focusing on both the pocket floor and internal walls, taken parallel and normal to the fiber orientation. Figure 12 illustrates the results in the form of box-and-whisker plots in which outliers arising from measurement errors were removed to ensure reliable data. In each plot, the whiskers mark the maximum and minimum values, the horizontal line inside the box represents the median, the cross denotes the arithmetic mean, and the box spans the 25th to 75th percentile range.

For the pocket floor, roughness measurements along (PF) and normal (NF) to the fiber orientation showed that tool T3 yielded superior surface finishes in both directions, recording (1.36 ± 0.03) μm parallel to the fibers and (1.48 ± 0.05) μm perpendicular to them. Tool T2 performed well across fiber orientations, achieving (1.60 ± 0.03) μm parallel and (1.59 ± 0.08) μm perpendicular. Interestingly, tool T6 provided a comparable roughness of (1.56 ± 0.12) μm perpendicular to the fibers but showed significantly higher values (2.28 ± 0.08) μm along the fibers, indicating directional sensitivity. Tool T4 recorded the highest roughness parallel to the fibers (3.45 ± 0.15) μm, while tool T5 recorded the poorest roughness perpendicular to the fibers (4.26 ± 0.16) μm. These results highlight how tool geometry and coating influence surface quality, with T3 and T2 showing less sensitivity to fiber orientation, whereas T4, T5, and T6 exhibit more pronounced orientation-dependent behavior.

A similar trend emerged for the internal walls (PW perpendicular to the fibers, NW normal). Tool T3 again delivered the best surface finish, producing (1.50 ± 0.10) μm perpendicular to the fibers and (1.94 ± 0.07) μm parallel to them. Tool T1 performed well in the parallel orientation at (3.70 ± 0.10) μm but yielded the highest roughness of (6.20 ± 0.40) μm perpendicular to the fibers. Tool T5 was the weakest performer parallel to the fiber orientation at (6.50 ± 0.30) μm. These data underscore how each cutter interacts with the anisotropic structure of the GFRP. Tools T3 and T2 seem inherently more stable across orientations, whereas T1, T4, T5, and T6 exhibit marked sensitivities that are likely tied to variations in cutting-edge geometry.

**Figure 13:**
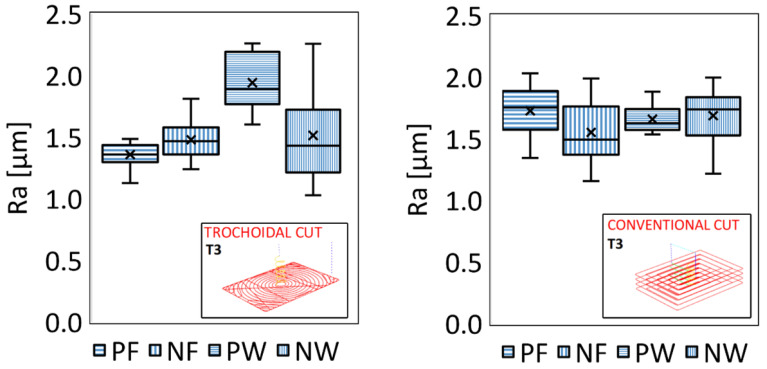
Comparison of Ra results for trochoidal (**left**) and conventional milling strategies (**right**) with tool T3.

A comparative assessment using tool T3 in both trochoidal and conventional milling (Figure 13) showed that well-optimized conventional milling can nearly match and occasionally surpass the roughness results obtained by trochoidal milling. Conventional milling with T3 yielded (1.73 ± 0.06) μm parallel to the fibers, (1.56 ± 0.07) μm perpendicular to the fibers, (1.69 ± 0.06) μm perpendicular to the walls, and (1.66 ± 0.03) μm parallel to the walls. While trochoidal milling is often favored for better management of cutting forces and heat generation, its circular tool path segments may inadvertently induce repetitive tool engagement or small-amplitude vibrations, potentially leaving a slightly rougher microtopography in the presence of abrasive glass fibers. In contrast, conventional milling follows more linear tool paths, which may stabilize chip formation and reduce the likelihood of over-scrubbing or chatter. As a result, under carefully tuned parameters, conventional milling can produce surface qualities comparable to or better than those of advanced adaptive techniques, especially when using a robust tool such as T3.

### 3.3. Dimensional Accuracy and Burr Formation

All dimensional and burr-related measurements reported in this study are presented with a probability of 68%, reflecting one standard deviation around the mean. Figure 9 illustrates the part with a rectangular slot in the center, highlighting the measured parameters *a*, *b*, and *c*. To reduce the dimensional errors as much as possible, constant cutting conditions were maintained for each tool, and a careful measurement procedure was followed before the machining process. Specifically, each tool was measured on a Heidenhain TT160 touch probe (Heidenhain, Schwenningen, Germany) to determine its radius and length offsets, ensuring consistent tool positioning and minimizing any discrepancies arising from tool-setup variations.

A summary of the dimensional measurements (*a*, *b*), their respective deviations Δ*a* and Δ*b*, and burr sizes (*c*) for the various tools and milling strategies is provided in Table 5. A comparison of pocket dimensions following trochoidal milling shows that most tools exhibit deviations from the nominal values on the order of tenths of a millimeter. Among these, tool T6 achieved the best outcomes, producing a long-side length of (59.890 ± 0.014) mm and a short-side length of (45.94 ± 0.03) mm. Tool T3 followed closely, measuring (59.890 ± 0.010) mm for the longer side and (45.913 ± 0.012) mm for the shorter side. In contrast, tool T1 fared worst, with (59.778 ± 0.019) mm on the long side and (45.770 ± 0.019) mm on the short side. Conventional milling proved more effective in maintaining dimensional accuracy, maintaining deviations within a mere hundredth of a millimeter (long side = 59.965 ± 0.004 mm, short side = 46.023 ± 0.008 mm).

Regarding burr formation, the smallest burrs—and consequently the cleanest cut edge—were produced by tool T4 at 0.49 mm, followed by tool T5 at 0.63 mm. Tool T6 exhibited the largest burrs (16.34 mm), suggesting less effective fiber shearing or higher levels of material pull-out. Conventional milling with tool T3 did not substantially reduce burrs—measuring 3.67 mm—outperforming some trochoidal results (e.g., T1 or T6) but lagging behind T4 and T5. A general observation is that tools featuring fewer flutes (T4, T5) tend to generate smaller burrs, likely owing to more aggressive cutting-edge geometries and efficient chip evacuation.

Tabulated comparisons of *a* and *b* and their respective deviations, Δ*a* and Δb, reinforce these observations. Although trochoidal milling can yield highly accurate dimensions with certain tools (for instance, T3 provided 59.890 ± 0.010 mm for *a*), the results varied depending on the specific tool geometry. Burr formation, denoted by the variable *c*, remained minimal in the trochoidal mode for T4 and T5, whereas T6 produced extensive burrs. Conventional milling with T3, on the other hand, showed excellent dimensional accuracy but a moderate burr size (3.67 mm).

### 3.4. Chip Size Distribution

**Figure 14:**
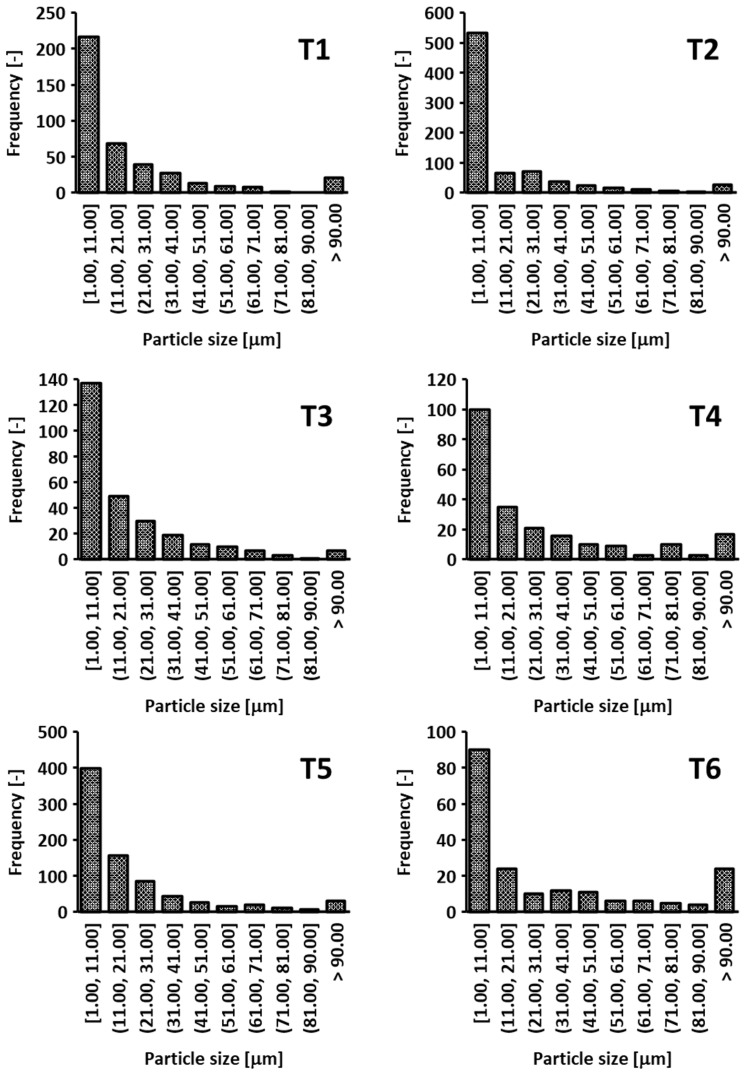
Comparison of chip size distribution for different tools during GFRP milling.

In assessing the overall quality of GFRP milling, the size distribution of the generated chips provides valuable insights into the material removal mechanisms and potential hazards (e.g., respiratory risks). After each cutting test, the loose chips were collected and placed on the glass stage of the digital imaging device. Using a reference scale (etalon) in each image, the particle size was identified using ImageJ software (version 1.52u). Figure 14 presents histograms illustrating the chip size distribution for tools T1–T6 during trochoidal milling. In all cases, the largest proportion of particles fell within the [1,11] μm range, with steadily decreasing frequencies at larger sizes. Tool T2 showed the highest overall chip count, indicating a more aggressive cutting action and potentially faster tool wear, whereas tools T1 and T5 produced a large quantity of very fine chips. In contrast, T3 and T6 generated lower frequencies in the smallest size interval, suggesting somewhat less intensive fiber fracture. Tool T4 showed a more balanced distribution of chip sizes, spanning fine to medium ranges.

**Figure 15:**
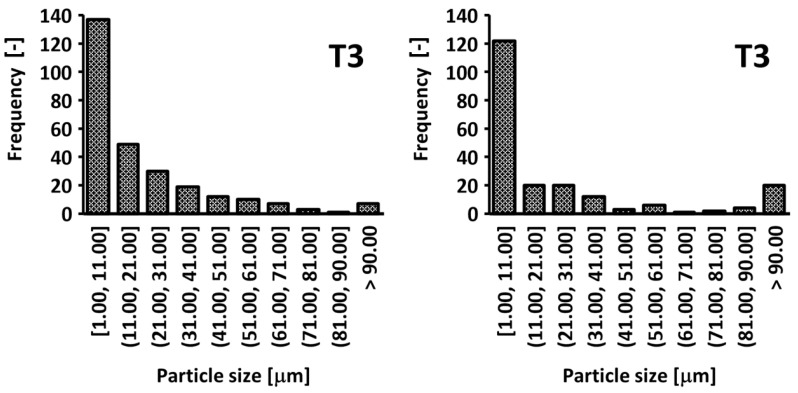
Chip size distributions for trochoidal (**left**) and conventional milling (**right**) using tool T3.

Figure 15 compares trochoidal and conventional milling for tool T3. Both strategies predominantly yield chips smaller than 11 µm; however, trochoidal milling produces a slightly wider range of sizes, including a modest increase in particles larger than 41 µm. In turn, conventional milling results in a narrower distribution with slightly fewer small chips. Although chip size measurements do not directly factor into the final ranking of tools, they are key to evaluating the risk of delamination (potentially signaled by higher counts above 90 µm) and ensuring operator safety (smaller particles below 11 µm require effective dust extraction). Hence, the choice of tool and milling strategy should also consider chip fragmentation characteristics to achieve an optimal balance between surface quality, productivity, and workplace health.

### 3.5. Tool Wear

**Figure 16:**
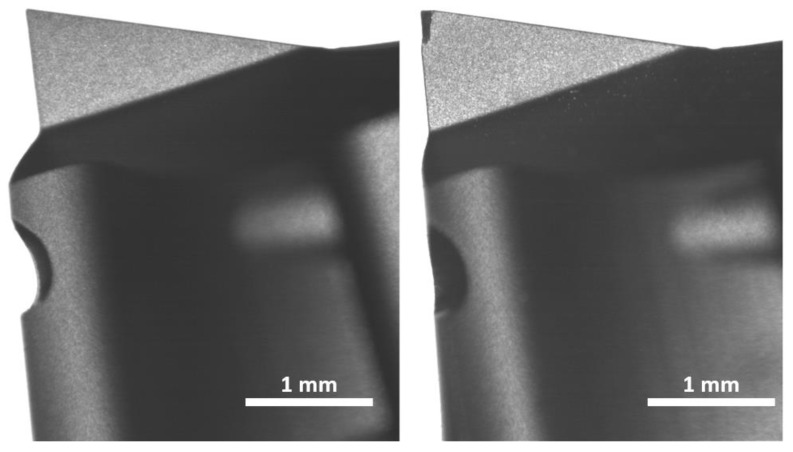
Tool T1 before (**left**) and after (**right**) trochoidal milling shows pronounced wear on the cutting edges.

Tool wear was assessed according to the methodology described in the Materials and Methods Section. Under trochoidal milling, tools T2, T3, and T5 showed minimal or negligible wear, indicating their suitability for long-duration GFRP machining using this strategy. In contrast, tool T6 exhibited moderate wear, whereas tools T1 (Figure 16) and T4 exhibited significant wear, suggesting limited viability in extended or high-volume production. Micrographic analyses confirmed pronounced flank wear and flute damage in T1 and T4, aligning with their lower overall scores in the subsequent tool ranking.

The wear levels were classified based on flank wear measurements as follows:

- Low wear: less than 50 µm;
- Moderate wear: 50–100 µm;
- High wear: above 100 µm.

Under trochoidal conditions, T1 and T4 fell into the high-wear bracket, whereas T2 and T5 exhibited moderate wear. T3 and T6 were consistently robust and displayed low wear. For conventional milling, only tool T3 was evaluated and maintained low wear, indicating that conventional machining may be less demanding on the cutting edges than the intermittent tool engagement characteristic of trochoidal milling.

This wear behavior directly influenced the final scoring, in which tool T3 achieved the highest overall performance, partly owing to its resilience against abrasive GFRP fibers. Although T1 and T4 performed adequately in certain metrics (e.g., specific force levels or burr formation), their rapid wear under trochoidal conditions contributed to notably lower overall ratings.

## 4. Cost Analysis

To quantify the economic feasibility of the different milling strategies and tools, a cost model was developed that consolidates three cost components—tool, machining, and surface quality—into a single measure of overall cost (*C_total_*). The total cost is expressed as follows:

*C_total_* = *C_tool_* + *C_machining_* + *C_quality_*, (1)

where *C_tool_* is the cost of the cutting tool, *C_machining_* represents the operating expense of the machine, and *C_quality_* reflects any finishing or quality-related costs (e.g., dealing with high roughness or extensive burr formation).

The machining cost is expressed as follows:

*C_machining_* = *R_machine_* × (*T_machining_* + *T_non_cutting_*), (2)

where *R_machine_* is the machine’s hourly or per-minute rate, *T_machining_* is the active cutting (in-cut) time, and *T_non_cutting_* includes rapid movements, tool lifts, and other ancillary motions.

The surface-quality cost is expressed as follows:

*C_quality_* = *k_finishing_* × Σ(*S_i_* × *Ra_i_*), (3)

where *k_finishing_* is a coefficient estimating the expense of reaching or improving a given surface finish, *S_i_* denotes the area of the *i*th machined surface (e.g., pocket floor or walls), and *Ra_i_* is the measured roughness in that area.

To compare the total costs across all tools and milling modes, a relative cost index (*C_ROI_*) was defined as follows:

*C_ROI,i_* = (*C_total,i_*/min(*C_total_*)) × 100, (4)

A value of 100% indicates the most cost-effective scenario (lowest overall *C_total_*), and any value above 100% signifies a percentage increase relative to the baseline. Table 6 presents the *C_ROI_* results for the six tools (T1–T6) under trochoidal conditions alongside the conventional operation with tool T3.

Evidently, tool T3 in the trochoidal mode achieves the lowest total cost, normalized to 100%. In contrast, certain other trochoidal configurations, such as T1, T4, and T5, exhibit substantially higher *C_ROI_* values, reflecting elevated tool or machining costs. Notably, T3 in conventional milling (*C_ROI_* = 114.53%) remains more economical than several other trochoidal options, illustrating that while trochoidal milling can reduce cycle times, its full cost benefit depends on how tool wear, machine usage, and final surface requirements are combined in practice.

A potential method to combine the advantages of trochoidal and conventional milling is to use trochoidal roughing, followed by a conventional finishing pass. In this study’s framework, the hybrid approach was approximated by leaving a 1 mm stock allowance during the trochoidal stage and then applying conventional cutting parameters with tool T3 to bring the part to final dimensions. Although these hybrid parameters were not experimentally optimized for distinct roughing and finishing regimes, a preliminary estimate based on existing machining data provides an initial illustration of the cost implications.

- Estimated process times: roughing cycle (trochoidal) ~3 min 36 s; finishing pass (conventional) ~1 min 58 s;
- Calculated ROI: 114.54%, effectively on par with tool T3 in the pure conventional mode (114.53%).

This outcome suggests that under the unaltered cutting parameters from single-mode tests, hybridizing the process offers no immediate cost advantage over purely conventional T3 milling. In practice, however, hybrid milling typically employs more aggressive roughing parameters, potentially reducing the roughing cycle time further at the expense of higher tool wear. Conversely, the subsequent finishing pass might preserve or enhance the surface quality and dimensional accuracy, possibly reducing quality-related costs.

Because the present study focuses on single-mode comparisons, we have not experimentally validated the hybrid surface outcomes (e.g., burr formation and roughness) under fully optimized dual-parameter sets. Nonetheless, our initial findings indicate that a hybrid approach can remain cost-competitive while potentially allowing for separate fine-tuning of the roughing and finishing stages. Future work will include dedicated trials that measure tool wear, cutting forces, and final surface quality in a purpose-optimized hybrid sequence to ascertain whether additional refinements can yield meaningful gains in both productivity and part quality.

## 5. Discussion and Tool Scoring

The findings of this study demonstrate that trochoidal (adaptive) milling can be highly effective in producing pockets in GFRP, particularly when combined with an appropriately matched cutting tool. This observation is consistent with earlier reports on composite machining, which suggest that minimizing radial engagement and dynamically adjusting feed rates—key features of adaptive milling—tend to reduce cutting forces and improve the surface finish. In line with these studies, the data herein confirm that advanced tool paths help mitigate fiber pullout and excessive burr formation, although some tool geometries (e.g., T1 and T4) remain susceptible to rapid wear under repetitive engagement.

A point-based scoring scheme was employed to objectively compare the six tools and determine their overall suitability. Each tool received a fixed number of base points in each category (10 points for the best performance), which were then multiplied by a weighting coefficient representing the practical significance of that category. All criteria, except tool wear, were derived from the quantitative data reported in the previous sections: cutting forces, surface roughness from all measured locations, dimensional deviations from the nominal geometry, and maximum burr size. These values were normalized and converted into point scores. Tool wear was the only category evaluated categorically, with three discrete wear states (low, moderate, and high) assigned numeric factors of 1.0, 0.5, and 0.33, respectively. As shown in Table 7, the importance of each criterion is assigned as follows:

- Surface quality (40%)—prioritized owing to stringent finish requirements in GFRP applications;
- Cutting forces (20%)—lower forces imply improved tool life and energy efficiency;
- Dimensional accuracy (20%)—ensures that the final parts remain within specified tolerances;
- Burr formation (10%)—important but assigned a lesser weight compared to surface finish and accuracy. Although burr formation is undesirable, it can typically be removed through additional post-processing, requiring extra time and effort;
- Tool wear (10%)—a crucial factor for tool cost and replacement intervals, yet also with relatively lower priority than surface finish or dimensional control. (Note: Even a tool experiencing noticeable wear can still be used to machine GFRP, albeit often at the expense of one or more performance metrics.)

A weighted scoring system emphasizing the surface quality, cutting forces, dimensional accuracy, burrs, and tool wear was used to evaluate six different end mills. Table 5 consolidates these scores, highlighting tool T3 as the top-performing solution in trochoidal milling. It consistently showed low cutting forces, high-quality surfaces, and minimal wear, in line with the hypothesis that sharper geometries and stable coatings are advantageous for fiber-reinforced plastics. Tool T2 also proved viable, although slightly higher wear rates and cost trade-offs may limit its attractiveness for large-scale operations. The mid-range performance of tools T5 and T6 indicates the potential for acceptable outcomes, where cost-effectiveness takes precedence over maximum precision. In contrast, tools T1 and T4 displayed weaker results overall, with severe wear or inconsistent accuracy that undermines their use for extended trochoidal milling in GFRP.

From a broader manufacturing perspective, these observations reinforce the importance of matching the tool geometry, coating, and milling strategy to prevent the typical pitfalls of composite machining: delamination and burr formation, rapid tool wear, and compromised dimensional tolerances. As observed in prior investigations of both carbon- and glass-fiber composites, even a worn tool can continue cutting GFRP, albeit with elevated forces and declining surface quality. Industries demanding particularly tight tolerances (e.g., aerospace) may, therefore, favor conventional or hybrid milling approaches, accepting longer cycle times in exchange for higher dimensional precision. Another promising approach combines both trochoidal and conventional milling in a single workflow; trochoidal milling can be used for efficient roughing and bulk material removal, as noted by Chang [64], followed by a finishing pass using a suitable GFRP tool under conventional milling. This hybrid strategy takes advantage of adaptive tool paths for rapid roughing while leveraging conventional methods for final dimensional control and surface refinement. Ultimately, the choice of strategy—purely adaptive, purely conventional, or a combination of the two—depends on the specific part requirements, cost constraints, and target surface quality.

The challenges encountered when milling GFRP extend beyond this study. Heat buildup emerges from friction at the tool–workpiece interface; once local temperatures surpass the resin’s softening point, the risk of fiber–matrix debonding and accelerated tool wear rises considerably [65]. Vibration-induced effects further compound the problem, because GFRP’s anisotropy fosters unstable cutting forces that trigger chatter and degrade surface integrity. Moreover, fiber orientation plays a decisive role in the mechanisms of material removal, frequently impacting delamination and tool life [66]. Adding to the complexity, moisture absorption can alter thermal and mechanical properties; although not tested here, prior studies suggest that elevated moisture levels can exacerbate heat and vibration issues by modifying the composite’s stiffness. Cooling methods (e.g., MQL, flood cooling) may help, but controlling temperature and chip evacuation simultaneously often proves challenging [67]. Subsurface damage, typically manifested as micro-cracking or hidden delamination, warrants close scrutiny in load-bearing applications, since small flaws can propagate over time [65]. Lastly, coating properties on the cutting tool can affect chip evacuation and heat distribution, emphasizing the importance of selecting or developing coatings tailored to the abrasive character of GFRP [66]. Addressing these multifaceted issues will remain a key priority in future investigations, where more extensive parameter studies and advanced characterization techniques (e.g., non-destructive testing) can better quantify and mitigate each source of damage.

These conclusions suggest several avenues for future research. For instance, sensor-based adaptive machining systems can be introduced to optimize tool engagement in real time, further extending tool life and maintaining consistent cutting forces. Exploring more advanced tool materials (e.g., polycrystalline diamond or specialized ceramic coatings) under adaptive paths may also clarify the limits of wear resistance in high-abrasion environments. Finally, systematically varying fiber orientations and resin types could offer broader insights into how geometry-specific factors affect cutting performance, paving the way for more robust and cost-effective solutions for GFRP and other composite machining applications.

## 6. Conclusions

This study demonstrates that trochoidal (adaptive) milling offers an efficient and practical strategy for pocket machining in GFRP, particularly when the cutting tool possesses both a robust coating and a low helix angle capable of resisting the abrasiveness of the material. The results confirm that reducing radial engagement and dynamically adjusting cutting parameters markedly diminish cutting forces, improve surface finish, and extend tool life, aligning with prior investigations on composite machining. At the same time, GFRP’s anisotropy and the fiber pull-out tendencies of GFRP require careful selection of the milling strategies and tool geometries.

The following key findings highlight the viability and performance of GFRP machining using both trochoidal and conventional milling approaches:

- Coated tools featuring a low helix angle proved optimal for balancing the surface quality, dimensional accuracy, and minimal wear. In this study, a four-tooth cutter with a 10° flute helix and aluminum-oxide coating achieved the best overall score (7.22 out of 10), indicating superior performance in both trochoidal and conventional cutting;
- Proper tool selection and process parameters can yield surface roughness levels of approximately (1.36 ± 0.03) μm on the pocket floor and (1.50 ± 0.10) μm on side faces, comparable to finishes generally associated with more easily machined materials;
- Conventional milling provides a tighter range of machining tolerances and can deliver 25%-lower RMS cutting forces than trochoidal milling, even at the expense of higher force maxima and a longer cycle time;
- Trochoidal milling exhibits shorter cycle times (approximately 23% faster than conventional milling of the same geometry), up to a 66%-higher material removal rate (MRR), more uniform force distribution, and an enhanced ability to handle higher throughput;
- Conventional milling achieves superior dimensional control and can generate higher-quality surfaces in fiber-sensitive regions, making it particularly useful for parts that require strict tolerances or refined finishes;
- In both strategies, the majority of chips measured below 11 μm, with trochoidal milling producing a broader range of particle sizes and a modest increase in chips above 41 μm;
- The combination of trochoidal roughing and conventional mill finishing has emerged as a promising approach: the initial adaptive phase rapidly removes bulk material under stable force conditions, while the subsequent conventional pass refines accuracy and surface quality in fiber-rich or tolerance-critical areas.

In summary, these results confirm the viability of adaptive milling for GFRP pocketing, provided that the cutting tool is designed to accommodate frequent, high-load engagements and a low helix angle. Meanwhile, conventional milling remains essential for achieving final-dimensional integrity and enhanced surface finish in precision applications. Embracing a hybrid strategy—rapid roughing via trochoidal cuts, followed by a conventional finishing pass—may deliver the most comprehensive benefits, namely, accelerated throughput, accurate features, and extended tool life. The insights gained highlight the potential for continued refinement of composite machining strategies, particularly through further exploration of tool geometries, coatings, and sensor-driven adaptive control.

## Figures and Tables

**Table 1:** Characteristics of dust particles generated during composite machining.

Composite Type	Machining Technology	Dust Particle Size	Reference
CFRP	Trimming (edge milling)	Most particles < 10 μm, ~85% < 2 μm	[54]
CFRP	Cutting, drilling, grinding	Respirable fibers < 3 μm, concentration up to 8.3 × 10^5^ fibers/m^3^	[55]
CFRP	Trimming (edge cutting)	Dust < 10 μm	[56]
CFRP	Dry CNC milling	0.5–10 μm (majority ~0.5 μm)	[57]
CFRP and GFRP	Drilling, grinding, milling	CFRP: min. ~0.1 μm, GFRP: min. ~0.05 μm, fiber length 15–60 μm	[58]
CFRP	Dry milling	1.7–99 μm, majority < 10 μm, smallest ~1.7 μm	[59]

**Table 2:** Specifications of cutting tools for machining GFRP material.

Specification	T1	T2	T3	T4	T5	T6
Cutting diameter	6.0 mm	6.0 mm	6.0 mm	6.0 mm	6.0 mm	6.0 mm
Max. depth of cut in feed direction side	18.0 mm	12.0 mm	18.0 mm	18.0 mm	18.0 mm	35.0 mm
Overall length	70 mm	65 mm	65 mm	65 mm	65 mm	75 mm
Flute helix angle	15.0 °	20.0 °	10.0 °	25.0 °	25.0 °	46 °
Corner radius	0 mm	0.5 mm	0.2 mm	0 mm	0 mm	0 mm
Corner chamfer angle	0 °	0 °	0 °	0 °	0 °	45 °
Face cutting edge count	2	4	4	2	10	4
Peripheral cutting edge count	5	4	4	10	10	4
Coating	DURA *	DURA *	DURA *	DURA *	DURA *	NXT *
Supplier	Seco	Seco	Seco	Seco	Seco	Seco
Designation	860060Z5.0-DURA	840060R050Z4.0-DURA	880060R020Z4.0-DURA	870060.0-DURA	871060.0-DURA	JS514060D4C.0Z4-NXT
Intended application	Slot + side (sandwich/honeycomb)	Slot + side, compressive design (layered composites)	Slot + side, low helix (thermosets/thermoplastics)	Multi-tooth router (2 face edges), side/slot/ramping	Multi-tooth router (10 face edges), high-volume removal	Universal corner cutter, deep side milling

* DURA refers to aluminum-oxide coating, and NXT refers to TiAlN coating.

**Table 3:** Cutting conditions for trochoidal and conventional milling.

Parameter	Units	Trochoidal	Conventional
Cutting speed	[m/min]	220	145
Spindle speed	[1/min]	11 671	7 692
Feed rate	[mm/min]	2652	1000
Axial depth of cut	[% of diameter]	200	25–50
Axial depth of cut	[mm]	12	1.5–3.5
Radial depth of cut	[% of diameter]	7	30
Radial depth of cut	[mm]	0.42	1.8
Chip area	[mm^2^]	5.04	6.3
Material removal rate (MRR)	[cm^3^/min]	13.37	2.7–6.3

**Table 4:** Cutting and specific cutting forces.

Tool	*Fx* RMS	*Fx* Max.	*Fy* RMS	*Fy* Max.	*Fz* RMS	*Fz* Max.	*Kc* RMS	*Kc* Max.
	[N]	[N]	[N]	[N]	[N]	[N]	[N/mm^2^]	[N/mm^2^]
Trochoidal								
T1	19.82	91.01	29.73	107.20	5.73	25.19	5.90	21.27
T2	21.68	105.80	29.24	118.80	7.92	48.99	5.80	23.57
T3	19.09	116.20	26.60	141.00	4.70	28.44	5.28	27.98
T4	23.28	123.40	28.34	116.90	11.51	51.36	5.62	24.48
T5	25.63	93.95	23.04	90.69	12.66	46.78	5.09	18.64
T6	19.15	105.90	30.25	99.96	18.39	101.80	6.00	21.01
Conventional								
T3	19.59	90.04	24.96	144.50	4.60	78.72	3.96	22.86

**Table 5:** Results of dimensional accuracy and burr formation measurements.

Tool	*a* [mm]	Δ*a* [mm]	*b* [mm]	Δ*b* [mm]	*c* [mm]
Trochoidal					
T1	59.778 ± 0.019	0.222	45.770 ± 0.019	0.230	11.6
T2	59.845 ± 0.018	0.155	45.80 ± 0.04	0.200	5.19
T3	59.890 ± 0.010	0.110	45.913 ± 0.012	0.087	8.60
T4	59.818 ± 0.017	0.182	45.853 ± 0.015	0.147	0.49
T5	59.850 ± 0.017	0.150	45.82 ± 0.03	0.180	0.63
T6	59.890 ± 0.014	0.110	45.94 ± 0.03	0.060	16.34
Conventional					
T3	59.965 ± 0.004	0.035	46.023 ± 0.008	−0.023	3.67

**Table 6:** Relative cost index *C_ROI_* for tool–strategy combinations.

Tool	Milling Strategy	*C_ROI_* [%]
T1	Trochoidal	179.53
T2	Trochoidal	131.09
T3	Trochoidal	100.00
T4	Trochoidal	233.92
T5	Trochoidal	238.96
T6	Trochoidal	135.07
T3	Conventional	114.53

**Table 7:** Weighted tool scoring for GFRP milling tools.

Tool	T1	T2	T3	T4	T5	T6	T3
Strategy	Trochoidal						Conventional
Surface quality	0.88	1.63	2.80	0.97	0.84	1.39	3.14
Specific cutting force	1.75	1.58	1.33	1.52	2.00	1.77	1.63
Dimensional accuracy	0.20	0.23	0.42	0.25	0.26	0.42	1.31
Burr formation	0.04	0.09	0.06	1.00	0.78	0.03	0.13
Tool wear	0.33	1.00	1.00	0.33	1.00	0.50	1.00
Total score	3.21	4.53	5.61	4.08	4.87	4.12	7.22

Table values reflect weighted scores, as described above. A higher number is better.

## Data Availability

The original contributions presented in this study are included in the article. Further inquiries can be directed to the corresponding authors.

## Appendix A

Below is a table that includes the mean (*μ*) and standard deviation (*s*) of the surface roughness (Ra) for four measured areas (PF, NF, NW, PW) across six tools (T1–T6) in trochoidal milling and one tool (T3) in conventional milling:

**Table A1:** Ra [µm] for different tools, measured areas, and cutting strategies (mean ± std. dev.).

Tool	T1	T2	T3	T4	T5	T6	T3
Parameter	Trochoidal						Conventional
Ra-PF (*μ*)	2.356	1.598	1.357	3.454	2.901	2.278	1.726
Ra-PF (s)	0.111	0.094	0.099	0.556	0.514	0.271	0.213
Ra-NF (*μ*)	2.227	1.588	1.478	3.631	4.256	1.564	1.550
Ra-NF (s)	0.426	0.267	0.168	0.830	0.593	0.442	0.260
Ra-PW (*μ*)	3.655	3.136	1.941	4.806	6.457	3.652	1.686
Ra-PW (s)	0.350	1.059	0.220	0.492	0.819	0.316	0.213
Ra-NW (*μ*)	6.172	3.344	1.513	5.556	4.149	3.895	1.657
Ra-NW (s)	1.133	0.414	0.370	1.721	0.706	0.977	0.105

PF = pocket floor (parallel), NF = pocket floor (normal), NW = pocket wall (normal), PW = pocket wall (parallel).
